# Coenzyme Q10 and the Blood–Brain Barrier: An Overview

**DOI:** 10.3390/jcm14082748

**Published:** 2025-04-16

**Authors:** David Mantle, Iain Hargreaves

**Affiliations:** 1 Pharma Nord (UK) Ltd., Morpeth NE61 2DB, UK; 2 School of Pharmacy and Biomolecular Sciences, Liverpool John Moores University, Liverpool L3 3AF, UK; i.hargreaves@ucl.ac.uk

**Keywords:** neurological disorders, mitochondrial dysfunction, blood–brain barrier, blood CSF barrier, coenzyme Q10, intranasal drug delivery

## Abstract

Mitochondrial dysfunction is a common factor known to be involved in the pathogenesis of a number of neurological disorders, including Parkinson’s disease, Alzheimer’s disease, and amyotrophic lateral sclerosis. Given the importance of coenzyme Q10 (CoQ10) in promoting normal mitochondrial function, and the deficiency of CoQ10 reported in such neurological disorders, there is a rationale for investigating the potential therapeutic role of supplementary CoQ10. However, while there is evidence for the efficacy of CoQ10 supplementation in animal models of the above disorders, randomised controlled clinical trials supplementing CoQ10 in PD, AD, or ALS have had disappointing outcomes. This in turn may be a reflection of the current uncertainty as to whether CoQ10 can access the blood–brain barrier in human subjects. In an attempt to further elucidate the disparity in outcomes of such preclinical and clinical studies, in this article we have reviewed evidence from the peer-reviewed literature to establish the ability of CoQ10 to access the brain via the BBB.

## 1. Introduction

Coenzyme Q10 (CoQ10) is usually described as a vitamin-like substance, although by definition CoQ10 is not a vitamin, since it is synthesised by most tissues within the human body. CoQ10 has a number of functions of vital importance to various aspects of cell function, but particularly to normal mitochondrial function. Within mitochondria, CoQ10 has a key role as an electron carrier (from complex I and II to complex III) in the mitochondrial electron transport chain during oxidative phosphorylation. It is also involved (as a cofactor of the enzyme dihydroorate dehydrogenase) in the metabolism of pyrimidines, fatty acids, and mitochondrial uncoupling proteins, as well as in the regulation of the mitochondrial permeability transition pore. CoQ10 serves as an important lipid-soluble antioxidant protecting mitochondrial membranes from free radical-induced oxidative stress. CoQ10 exists in both oxidised (ubiquinone) and reduced (ubiquinol) forms, and the normal functioning of CoQ10 involves continual inter-conversion between these two forms [1].

Mitochondrial dysfunction is a common factor known to be involved in the pathogenesis of a number of neurological disorders, including Parkinson’s disease (PD), Alzheimer’s disease (AD), amyotrophic lateral sclerosis (ALS), and multiple system atrophy (MSA). There is evidence for deficiency of CoQ10 in the above disorders, for example in cerebral cortex from PD patients and cerebellar tissue from MSA patients [2]. Given the importance of CoQ10 in promoting normal mitochondrial function, and the deficiency of CoQ10 reported in the above neurological disorders, there is a rationale for investigating the potential therapeutic role of supplementary CoQ10.

However, while there is evidence for the efficacy of CoQ10 supplementation in animal models of the above disorders, randomised controlled clinical trials supplementing CoQ10 in PD, AD, or ALS have had disappointing outcomes. This in turn may be a reflection of the current uncertainty as to whether CoQ10 can access the blood–brain barrier in human subjects [3]. In an attempt to further elucidate the disparity in outcomes of such preclinical and clinical studies, in this article we have reviewed evidence from the peer-reviewed literature to establish the ability of CoQ10 to access the brain via the BBB.

## 2. Blood–Brain Barriers

The process by which supplemental CoQ10 is absorbed from the digestive tract to enter systemic blood circulation has been reviewed by Mantle and Dybring (2020) [4]. Once CoQ10 has entered the bloodstream, there are two barriers potentially limiting access of CoQ10 into the brain, namely the blood–brain barrier (BBB) and blood cerebrospinal fluid (CSF) barrier (BCSFB). The BBB and BCSFB are anatomically and functionally distinct, being comprised of completely different epithelial/endothelial components. Thus, the BBB forms the barrier between the blood and interstitial fluid within the brain parenchyma, whilst the BCSFB forms the barrier between the blood and CSF encompassing the surface of the brain.

The BBB is formed by the brain capillary endothelium, comprising two membranes in series, the luminal and abluminal membranes, which are separated by about 200 nm of endothelial cytoplasm. The capillary endothelial cells are supported (structurally and functionally) by pericytes embedded in the basement membrane and astrocytic endfeet; pericytes cover approximately 30% of brain capillaries, while the astrocytic endfeet cover approximately 99%, respectively. Owing to the presence of high resistance tight junctions within the brain capillary endothelium, the intercellular pores that exist in the endothelial barriers in peripheral organs are absent in the endothelial barrier in the brain. Furthermore, there is minimal fluid-phase pinocytosis in the brain capillary endothelium, and the absence of paracellular or transcellular channels within the BBB means that substances in the blood circulation gain access to brain interstitial fluid via only one of two mechanisms, diffusion through the BBB or carrier- or receptor-mediated transport through the BBB. Transmembrane diffusion is a non-saturable mechanism that depends on the substance melding into the cell membrane. A limited range of substances are able to access the BBB by transmembrane diffusion, provided they are lipophilic and have a molecular weight <400 Da [5].

A number of BBB carrier-mediated (saturable) transporters have been identified, including those for glucose, amino acids, carboxylic acids, and purine nucleosides. In general terms, these carriers are responsible for the transport of nutrients from the blood into the brain. However, these carriers can also enable the access of drugs into the brain; for example, L-DOPA is an effective treatment for Parkinson’s disease because this dopamine precursor is an amino acid analogue that is transported across the BBB via the amino-acid carrier. In receptor-mediated transport, a circulating ligand binds to a specific receptor expressed on the luminal membrane of the brain capillary endothelium that forms the BBB. Once bound to the ligand, the process of endocytosis is initiated with the formation of intracellular transport vesicles. In a process of transcytosis, the receptor-ligand-containing vesicles move across the cell to the basolateral side of the endothelial cells, where they are released. In this way, substances are able to cross the endothelium and enter the brain without disruption of the barrier properties. Using the latter route, substances such as insulin and iron are transported into the brain via insulin and transferrin receptors, respectively [6]. A novel method of accessing the BBB via receptors is the use of so-called Trojan horse technology. A BBB molecular Trojan horse is a monoclonal antibody raised against a receptor (such as the insulin or transferrin receptors), which accesses the BBB following receptor binding, at the same time delivering a pharmaceutical drug genetically fused to the antibody [7].

It is of note that the BBB is also the location for a number of efflux transporters of the so-called ABC (ATP-binding cassette) family, including P-glycoprotein, which is active against a diverse range of drugs [8]. these function as brain-to-blood efflux pumps preventing or retarding the entry of substances (including small lipophilic molecules) into the brain [9]. In contrast to the brain capillary endothelium, P-glycoprotein is not expressed at the choroid plexus, another factor in the increased permeability of the BCSFB compared to the BBB [10].

The BCSFB is formed by epithelial cells of the choroid plexus, which is leaky compared to the BBB, as reflected by the electrical resistance across these two barriers. The electrical resistance across the choroid plexus epithelial barrier is 26 ohm·cm^2^, while the electrical resistance across the endothelium of capillaries within brain parenchyma is estimated to be 8000 ohm·cm^2^, some 300-fold higher than the resistance across the BCSFB [11]. Virtually all small and large molecules in blood penetrate into CSF, at a rate inversely related to the molecular weight of the substance. Thus drug entry into the CSF compartment from blood should not be taken as an index of drug transport across the BBB, a common misconception. A similar misconception is that that a substance injected into the CSF compartment distributes to the inner parenchyma of the brain; substances injected into the CSF undergo rapid efflux to the blood compartment via bulk flow. Conversely, as noted above, the movement of a substance into brain tissue from the CSF occurs via diffusion. The differential rates of bulk flow and diffusion create a paradox that substances injected into the CSF distribute readily into blood and poorly to brain beyond the ependymal surface. The difference in permeability between the BBB and BCSFB is illustrated by the drug azidothymidine, which is rapidly transported across the choroid plexus epithelium and rapidly enters the CSF in humans; in contrast, there is negligible transport of azidothymidine across the BBB, as a consequence of active efflux transport processes at the latter [12].

There is some evidence that the BBB in rodents may be more accessible to given substances than the BBB in humans. Thus, Uchida et al. (2020) [13] reported that protein expression levels of transporters and receptors in the BBB of humans were remarkably smaller than those in the BBB of rats.

## 3. Intranasal Drug Delivery

There is a mechanism by which substances can be delivered directly into the brain, bypassing the BBB and BCSFB, namely the intranasal route. In addition to bypass of the BBB, other advantages of intranasal drug delivery include rapid onset of action, avoidance of hepatic first-pass metabolism, and good patient compliance.

The interior of the nose is divided into several regions, the olfactory and respiratory regions being the most important with regard to intranasal drug delivery. The first step in drug absorption from the nasal cavity is to cross the mucus layer. Small (<1 kDa) lipophilic molecules most easily pass through the mucus layer (CoQ10 MW 863 Da, highly lipophilic). The drug then needs to access the nasal epithelial layer, which can occur via transcellular diffusion, paracellular transport, endocytic vesicle-mediated transport, and carrier-mediated transport. Transcellular diffusion is the main mechanism of absorption and is the most suitable for lipophilic and low molecular weight molecules.

The olfactory and respiratory epithelia are innervated by the olfactory and trigeminal nerves, respectively, which provide a route for drugs to access the brain directly. The olfactory and trigeminal pathways are the only routes by which the brain is connected to the outside environment. Transport of drugs via these pathways can occur via two mechanisms, intracellular and extracellular. In the intracellular mechanism, the drug is taken up by neurons through a process of endocytosis, then transported (via endosomes) within the nerves to the pons region of the brainstem (and thence to the cerebrum and cerebellum) in the case of the trigeminal nerve, and to the olfactory bulb and frontal cortex in the case of the olfactory nerve. In the extracellular pathway, the drug is first absorbed into the lamina propria. From the lamina propria, the drug can be absorbed by local blood vessels or lymphatic vessels, but most of the drug is translocated through the perineural space, between olfactory ensheathing cells and olfactory nerve fibroblasts. This space leads to the subarachnoid space of the brain from where the substance can be further distributed. In both mechanisms, the drug travels through the cribriform plate, which separates the brain from the nasal cavity. The intracellular mechanism is relatively slow, and may take several hours for drugs to reach the brain. The extracellular mechanism is much faster, with drugs taking 2–3 min to reach the brain. It is of note that some types of virus, including the SARS-COV-2 virus (olfactory epithelium expresses the obligatory angiotensin-converting enzyme II receptor), can access the brain via transport along the olfactory or trigeminal nerves.

There are several obstacles that can limit absorption of drug through this pathway, such as poor drug permeability from the nasal mucosa, mucociliary clearance, enzymatic degradation of the drug, low drug residence time, and possible toxicity that the formulation or drug can produce on the nasal mucosa. In addition, when a drug is deposited in the nasal cavity, and depending on its residence time, a part of it can be absorbed and enter the systemic circulation due to the rich vasculature of the respiratory epithelium and reach the brain through the BBB. This is a secondary route of drug transport to the brain after nasal administration.

It should be noted that the nasal anatomy in animals can differ widely relative to humans. In particular, the ratio of surface area to luminal volume in the nasal cavity of a rat is very different from that in humans. The relatively larger dedication of olfactory mucosa to smell in rodents allows a particularly strong model for intranasal delivery, but a lower contribution of olfactory mucosa to smell in humans poses a challenge when interpreting drug efficacy results from rodent data.

## 4. CoQ10 Metabolism

Before discussion of the subject of CoQ10 and brain access in the following section of this article, it is of relevance to first consider some more general aspects of CoQ10 metabolism. Although considerable advances in the understanding of some aspects of CoQ10 metabolism have been made, there are still many aspects of CoQ10 metabolism that are poorly understood. Thus, much progress has been made in understanding the CoQ10 biosynthetic pathway; at least 10 enzyme-encoding genes are required for the biosynthesis of functional CoQ10. Many of the data relating to CoQ10 biosynthesis were initially obtained from studies in yeast, with deficiencies corresponding to the above genes denoted as CoQ1 to CoQ11 (the numbering refers to date order of identification). The biosynthesis of CoQ10 in yeast and man has subsequently been shown to be highly conserved [14].

However, the process by which CoQ10 is transported from its principal site of biosynthesis (in the inner mitochondrial membrane) to where it is required elsewhere within the cell (plasma membrane, endoplasmic reticulum, Golgi apparatus, peroxisomes, lysosomes) is not fully understood. In this regard, two possible CoQ10 transporters have been identified in yeast, namely Cqd1 and Cqd2 of the UBI family, responsible for the intracellular distribution of CoQ6 (the principal form of CoQ in yeast) [15]. It remains to be seen whether there is functional conservation between Cqd1 and Cqd2 and their putative human analogues. Another type of CoQ10 transporter of relevance to human CoQ10 metabolism was identified as saposin B, a lipid-binding protein, and CoQ10 complexed with saposin B has been identified in several types of human cells [16].

Another aspect of CoQ10 metabolism requiring further elucidation is the process by which exogenous CoQ10 accesses human cells from the bloodstream. It has been postulated that a high level of CoQ10 in the blood is required to drive CoQ10 into the cells of tissues; this in turn suggests a lack of specific transporters of CoQ10, with access to cells reliant on simple diffusion, which is slow and depends on the concentration gradient provided by a high circulating dose [3] With regard to the relative contributions of endogenous and exogenous CoQ10 to the body’s daily CoQ10 requirements, it is of note that the latter quantity has not been established.

In summary, CoQ10 is synthesised in most human tissues, including brain tissue; although the mechanism by which CoQ10 is synthesised in the brain is known, the process by which it is subsequently distributed within the cell, and that whereby exogenous CoQ10 accesses brain cells (whether from blood or CSF) require further elucidation. Patients with primary CoQ10 deficiency (resulting from mutations in genes directly involved in the CoQ10 biosynthetic pathway) typically present with muscular, renal, or neurological dysfunction, depending which of the genes has been affected. While patients with muscle or renal dysfunction may respond well to oral CoQ10 supplementation (providing the condition is identified sufficiently early), those with cerebral dysfunction typically do not respond in such a beneficial manner; the correction of CoQ10 deficiency in cultured fibroblasts or yeast models, but not in patients with neurological symptoms, suggests an insufficient uptake of exogenous CoQ10 across the blood–brain barrier [17].

## 5. CoQ10 and Brain Access

Studies using animal model systems of PD, AD, and ALS have shown promising evidence of benefit following supplementation with CoQ10. Thus, administration of CoQ10 has been shown to be beneficial in paraquat-induced or MPTP-induced murine models of Parkinson’s disease by improving behaviour, reducing oxidative stress, or preventing loss of dopamine [18]. Similarly, supplementary CoQ10 reduced oxidative stress and beta-amyloid deposition and improved cognitive performance in transgenic mouse models of Alzheimer’s disease [19]. However, as noted in the Introduction, randomised controlled clinical trials supplementing CoQ10 in PD, AD, or ALS have had disappointing outcomes. For example in ALS, although supplemental CoQ10 or its synthetic analogue MitoQ prolonged survival in a mouse model of ALS [20,21], a Phase II trial supplementing 2700 mg CoQ10/day for 9 months in 185 ALS patients found insufficient benefit to warrant a Phase III study [22]. It should be noted that the endpoints in the preclinical and clinical studies differed i.e., prolonged lifespan in animal models and functional decline in patients; however, significant benefit was demonstrable in the former studies, and not in the latter. Randomised controlled trials of supplementary CoQ10 in Parkinson’s disease patients have had similarly disappointing outcomes [23].

There are a number of factors that may contribute to the unsuccessful outcome of randomised controlled trials supplementing CoQ10, including the bioavailability of the supplement used [17]. However, the disparity in outcomes between preclinical and clinical studies may be a reflection of the differing ability of CoQ10 to access the BBB in rodents and in man. There is some evidence that supplementary CoQ10 can cross the blood–brain barrier in rodents. For example, oral administration of CoQ10 (200 mg/kg/day for 1–2 months) in 12–24-month-old rats resulted in a subsequent increase of 30–40% in cerebral cortex CoQ10 levels [24]. Similarly, supplementation with emulsified CoQ10 (150 uM for 7 days) in 15-month-old mice increased CoQ10 levels in brain mitochondria [25].However, whether CoQ10 can cross the blood–brain barrier in man has yet to be established, as further addressed in the following section.

No clinical studies were identified in which orally administered CoQ10 was shown directly to cross the BBB and access the human brain. Such studies would involve, for example, the use of the imaging of radiolabelled CoQ10, or the analysis of tissue biopsy samples, which for obvious ethical reasons, would not be possible. It has been suggested that in human subjects the reduced form of CoQ10 (ubiquinol) may be able to access the BBB, while the oxidised form of CoQ10 (ubiquinone) is not able to cross the BBB. Thus, following oral supplementation with ubiquinol, Mitsui et al. (2017) [26] reported increased levels of ubiquinol in the CSF of patients with multiple system atrophy (MSA); however, as noted in a previous section of this article, access of a substance into the CSF (presumably via the BCSFB) does not equate to access of the substance into the brain parenchyma. In this regard, another factor to consider is the difficulty in measuring the low levels (low nanomolar range) of CoQ10 present in CSF; only HPLC linked to electrochemical detection or mass spectrometry are sufficiently sensitive methods for this analysis ([27]; Table 1). Tentative reference ranges have been reported for CSF CoQ10 levels of 1.18–4.91 nM established from a patient cohort (*n* = 15) aged 9–18 years [28] and 5.7–9.0 nM established from a patient cohort (*n* = 17) aged 0.1–22 years [27], respectively. The different age ranges of the patient cohorts may have contributed to the disparity between the reference ranges; however, a study by Isobe et al. (2010) [29] reported no correlation between age and CSF CoQ10 status, although this study was undertaken in adults aged 65.8 ± 12.4 years (mean ± SD), and no investigation was undertaken in children. Therefore, in order to establish a more reliable and robust CSF CoQ10 reference range, further studies are required that evaluate the effects of age and gender, as well as the rostral–caudal gradient upon CSF CoQ10 status.

As noted above, there are essentially two ways for substances to access the BBB-diffusion and carrier/receptor-mediated transport. For access via diffusion, the substance must be a small lipophilic molecule, with a molecular weight of <400 Da. On this basis of molecular weight restriction, CoQ10 would be unlikely to cross the BBB via diffusion, in either ubiquinone or ubiquinol forms. With regard to carrier- or receptor-mediated transport, to date there have been no studies that have identified carriers/receptors for CoQ10 in the human brain. While there have been no studies unequivocally demonstrating the access of CoQ10 into the human brain, there is an interesting “indirect” study by [31] which showed (via 31P NMR) improved energy metabolism in the brains of patients with mitochondrial cytopathies following oral supplementation with CoQ10.

**Figure 1:**
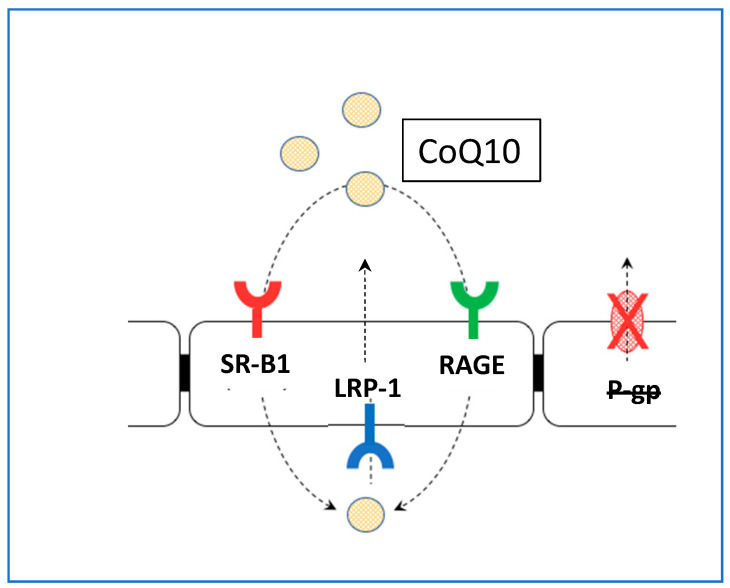
Schematic figure showing the transport CoQ10 across the BBB. No “net” CoQ10 entry toward brain side. Uptake by RAGE and SR-B1 is opposed by LRP-1/LDLR-mediated removal to blood, a major impediment to brain entry of CoQ10. **SR-B1:** Scavenger Receptor B1; **RAGE:** Receptor Advanced Glycation End-Products; **LRP-1:** Low Density Lipoprotein-Related Protein-1.

To further elucidate the mechanism through which CoQ10 may access the blood–brain barrier, using a model system based on porcine brain endothelial cells, Wainwright et al. (2020) [32] identified lipoprotein-associated CoQ10 transcytosis in both directions across the in vitro BBB. CoQ10 uptake via SR-B1 (Scavenger Receptor) and RAGE (Receptor for Advanced Glycation Endproducts) receptors was matched by efflux via LDLR (Low Density Lipoprotein Receptor) transporters, resulting in no “net” transport across the BBB (Figure 1).

When CoQ10 deficiency was induced in the model (using p-aminobenzoic acid), BBB tight junctions were disrupted and CoQ10 “net” transport to the brain side increased.

One method of attempting to access the BBB is the use of synthetic analogues of CoQ10 such as mitoquinone (MitoQ), a conjuagate of ubiquinone and the triphenylphosphonium cation, developed with the intention of improving blood–brain barrier penetration and mitochondria-specific targeting [33]. However, it should be noted that because the chemical structure of mitoquinone is different to that of CoQ10, the intracellular functions also differ. While CoQ10 and mitoquinone have antioxidant action in common, mitoquinone is not able to transfer electrons from Complex I to Complex III in the mitochondrial electron transport chain during oxidative phosphorylation; in addition, the role of mitochondrially targeted mitoquinone in the metabolism of other subcellular organelles such as lysosomes has yet to be established.

A further method of accessing the BBB is the use of nanocarriers. Several types of nanocarriers have been developed to deliver therapeutics. Nanocarriers are able to cross the BBB and specifically target the brain due to their nanoscale (100 to 1000 nm) dimensions [34]. Several studies using CoQ10 loaded nanocarriers have been reported, for example, in the prevention of ischaemia reperfusion injury during heart transplantation [35], but to date there have been no studies reported in which CoQ10 loaded nanocarriers have been used to access the BBB.

## 6. Summary

1. There are essentially two barriers preventing access of CoQ10 into the human brain, the BBB and BCSFB. The BCSFB is relatively leaky compared to the BBB, and access of CoQ10 (in either ubiquinone or ubiquinol form) into the CSF via the BCSFB does not equate to access of CoQ10 into the brain parenchyma—a common misconception.
2. There are essentially two ways for substances to access the BBB-diffusion and carrier/receptor-mediated transport. For access via diffusion, the substance must be a small lipophilic molecule, with a molecular weight of <400 Da. On the basis of this molecular weight restriction, Q10 would be unlikely to cross the BBB via diffusion, in either ubiquinone or ubiquinol forms. With regard to carrier or receptor-mediated transport, to date there have been no studies that have identified carriers/receptors for Q10 (in either ubiquinone or ubiquinol form) in the human brain.
3. No clinical studies were identified in which access of orally administered CoQ10 (in either form) across the BBB was directly demonstrated in humans.
4. The use of CoQ10 analogues such as mitoquinone and idebenone to access the BBB/mitochondria has been proposed; however, although these analogues have antioxidant action in common with CoQ10, they differ from CoQ10 in other aspects of intracellular function.
5. A possible mechanism for delivering CoQ10 into the human brain, which bypasses the BBB, is intranasal drug delivery. However, to date no clinical studies have been carried out to establish the efficacy and safety of this route for delivery of CoQ10 into the human brain. Boyuklieva et al. (2024) [36] have described the preparation of idebenone-loaded nanocomposite microspheres suitable for nasal administration. However, as noted in item 3 above, the intracellular function of idebenone differs from that of CoQ10.

## Figures and Tables

**Table 1:** Current methods available to detect CoQ10 in tissue, blood, and CSF.

Tissues	Method to Detect CoQ10	Reference
Muscle and other tissue including brain	HPLC UV detection at 275 nm HPLC linked to electrochemical detection	[30] [28]
Blood, plasma, and serum	HPLC UV detection at 275 nm HPLC linked to electrochemical detection	[30] [28]
Cerebrospinal fluid (CSF)	HPLC linked to electrochemical detection Liquid chromatography-mass spectrometry	[28] [27]
